# Hospital Triage System for Adult Patients Using an Influenza-Like Illness Scoring System during the 2009 Pandemic—Mexico

**DOI:** 10.1371/journal.pone.0010658

**Published:** 2010-05-14

**Authors:** Eduardo Rodriguez-Noriega, Esteban Gonzalez-Diaz, Rayo Morfin-Otero, Gerardo F. Gomez-Abundis, Jaime Briseño-Ramirez, Hector Raul Perez-Gomez, Hugo Lopez-Gatell, Celia M. Alpuche-Aranda, Ernesto Ramírez, Irma López, Miguel Iguala, Ietza Bojórquez Chapela, Ethel Palacios Zavala, Mauricio Hernández, Tammy L. Stuart, Margarita Elsa Villarino, Marc-Alain Widdowson, Steve Waterman, Timothy Uyeki, Eduardo Azziz-Baumgartner, for the Hospital Civil de Guadalajara, Fray Antonio Alcalde Emerging Respiratory Infections Response Team

**Affiliations:** 1 Hospital Civil de Guadalajara, Fray Antonio Alcalde, Guadalajara, Jalisco, México; 2 Instituto de Patología Infecciosa y Experimental, Centro Universitario Ciencias de la Salud, Universidad de Guadalajara, Guadalajara, Jalisco, México; 3 Dirección General de Epidemiología, México Ministry of Health, México City, Distrito Federal, México; 4 National Public Health Laboratory, México City, Distrito Federal, México; 5 Public Health Agency of Canada, Winnipeg, Manitoba, Canada; 6 United States Centers for Disease Control and Prevention, Atlanta, Georgia, United States of America; Duke University Medical Center, United States of America

## Abstract

**Background:**

Pandemic influenza A (H1N1) virus emerged during 2009. To help clinicians triage adults with acute respiratory illness, a scoring system for influenza-like illness (ILI) was implemented at Hospital Civil de Guadalajara, Mexico.

**Methods:**

A medical history, laboratory and radiology results were collected on emergency room (ER) patients with acute respiratory illness to calculate an ILI-score. Patients were evaluated for admission by their ILI-score and clinicians' assessment of risk for developing complications. Nasal and throat swabs were collected from intermediate and high-risk patients for influenza testing by RT-PCR. The disposition and ILI-score of those oseltamivir-treated versus untreated, clinical characteristics of 2009 pandemic influenza A (H1N1) patients versus test-negative patients were compared by Pearson's Χ^2^, Fisher's Exact, and Wilcoxon rank-sum tests.

**Results:**

Of 1840 ER patients, 230 were initially hospitalized (mean ILI-score = 15), and the rest were discharged, including 286 ambulatory patients given oseltamivir (median ILI-score = 11), and 1324 untreated (median ILI-score = 5). Fourteen (1%) untreated patients returned, and 3 were hospitalized on oseltamivir (median ILI-score  = 19). Of 371 patients tested by RT-PCR, 104 (28%) had pandemic influenza and 42 (11%) had seasonal influenza A detected. Twenty (91%) of 22 imaged hospitalized pandemic influenza patients had bilateral infiltrates compared to 23 (38%) of 61 imaged hospital test-negative patients (p<0.001). One patient with confirmed pandemic influenza presented 6 days after symptom onset, required mechanical ventilation, and died.

**Conclusions:**

The triaging system that used an ILI-score complimented clinicians' judgment of who needed oseltamivir and inpatient care and helped hospital staff manage a surge in demand for services.

## Introduction

The severity of seasonal influenza epidemics is unpredictable and influenced by the predominant circulating virus strains and level of immunity in the population [1]. During peak community influenza activity, hospitals and emergency rooms may be overwhelmed by patients presenting with influenza-like illness (ILI) and more severe disease [2], [3]. Illness attack rates may be higher among most age groups during pandemics than observed for seasonal influenza due to limited immunity among exposed populations [4]. The re-emergence of highly pathogenic avian influenza A (H5N1) virus among poultry with sporadic transmission to exposed persons and the resulting high mortality has stimulated global influenza pandemic preparedness [5].

Key features of pandemic influenza planning are developing strategies to meet expected increased demand for patient care, and how to allocate limited resources, including ventilators and critical care [6]–[9]. Guidance has been developed for clinical triage of patients with ILI, including special populations (e.g. children, pregnant women), during a pandemic [10]–[12]. A key clinical decision is determining which ill persons can be managed as outpatients and which require hospitalization. Scoring systems, with varying predictive power, have been developed to determine who will require hospitalization, need ICU care, require a ventilator, or is at high risk of death (e.g. CURB-65)[13]–[15].

The emergence of 2009 pandemic influenza A (H1N1) virus has presented a great challenge for clinicians throughout the world [16]. Overwhelming demand for medical care by patients with ILI and limited availability of oseltamivir necessitated that clinicians rapidly triage patients for outpatient care or hospital admission. These challenges are compounded by the need for early oseltamivir treatment of influenza patients for optimal efficacy [17]. At the Hospital Civil de Guadalajara, Fray Antonio Alcalde (HCGFAA), Mexico, clinicians from the Adult Infectious Diseases Unit used a modified ILI scoring system to systematically triage adult patients with respiratory complaints and determine who would be prioritized for hospitalization and antivirals. We describe this triaging system during the peak 2009 pandemic in Guadalajara (April–August, 2009).

## Methods

**Figure 1:**
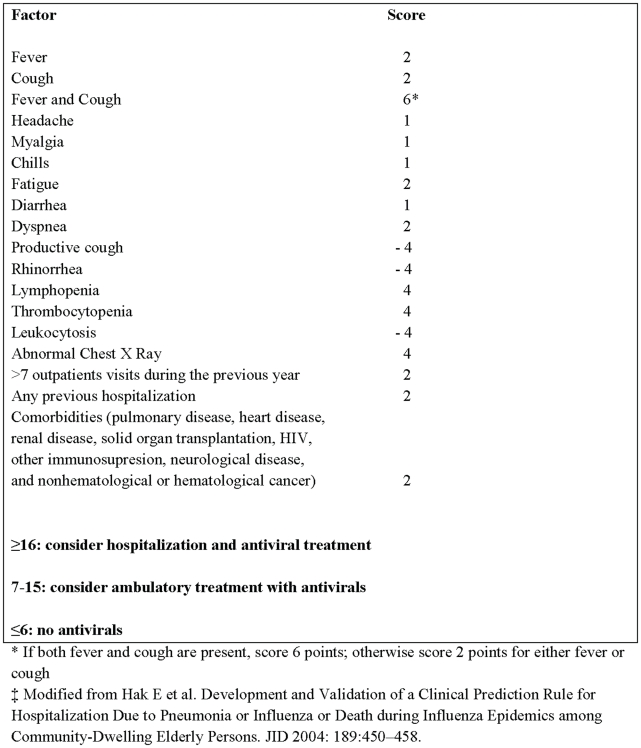
Influenza Scoring System at the Hospital Civil de Guadalajara during the (H1N1) pandemic 2009—Mexico_‡_.

HCGFAA is a 1000-bed tertiary care facility with a 30-bed infectious diseases unit. In response to high demand for emergency medical services among adult patients with acute respiratory complaints, infectious disease specialists implemented an ILI scoring system on April 25, 2009. This scoring system was adapted from a system developed by Hak et al in the United States for hospitalization decision-making among elderly patients with pneumonia or influenza during influenza epidemics [18]. In the emergency room (ER), a questionnaire was used to record patients' demographics, signs and symptoms, history of health care utilization, chronic medical conditions, laboratory, and radiology findings to calculate patients' ILI-scores (Figure 1). Clinicians used an ILI-score ≥16 (high-risk), their judgment of patients' severity of illness and proximity to the hospital to decided whether to admit the patient and treat them with oseltamivir. Patients with intermediate ILI-scores (7–15) were discharged from the ER, treated with oseltamivir and followed daily by phone for 10 days. Those with low ILI-scores (≤6) were discharged without antiviral treatment, and instructed to return if their symptoms worsened.

Nasal and throat swab specimens were collected from all high-risk and intermediate-risk patients. Swabs were combined in phosphate-buffered saline viral transport media and split into aliquots for influenza testing. One aliquot was tested by rapid diagnostic test (QuickVue Influenza Test, Quidel, San Diego, CA) and immunofluorescence at the hospital. A second aliquot was sent frozen at −70°C to the National Public Health (InDRE) laboratory in Mexico City. InDRE tested the samples with real-time RT-PCR (rRT-PCR) using a multiplex assay and 4 sets of primers (i.e. influenza A, universal swine, 2009 pandemic influenza A (H1N1), and a control for human genetic material) [19]. Each hospitalized patient had a chest x-ray and a chest CT scan performed at admission.

Clinicians prescribed standard doses of oseltamivir 75 mg BID for five days [17]. Hospitalized patients assessed to have severe illness received 150 mg of oseltamivir PO BID ×5 days, amantadine 300 mg PO BID ×10 days, broad spectrum antibiotics (e.g. linezolid), and paracetamol. Patients were discharged when afebrile and without dyspnea.

Patients' demographics, clinical presentation, treatments, and outcome data were entered into an SPSS database. The ILI-score, treatment, disposition, and virology results of triaged patients were compared by Pearson's Χ^2^, Fisher's Exact, Student t-tests, and Wilcoxon rank-sum tests.

The study was approved by the research ethics committee of the Hospital Civil de Guadalajara, Fray Antonio Alcalde and the final draft for publication was also approved by the research ethics committee of the Hospital Civil de Guadalajara, Fray Antonio Alcalde. Investigators kept the datasets in password protected systems and presented data without identifiers to protect the anonymity of case-patients.

## Results

### Disposition of Triaged Patients

**Figure 2:**
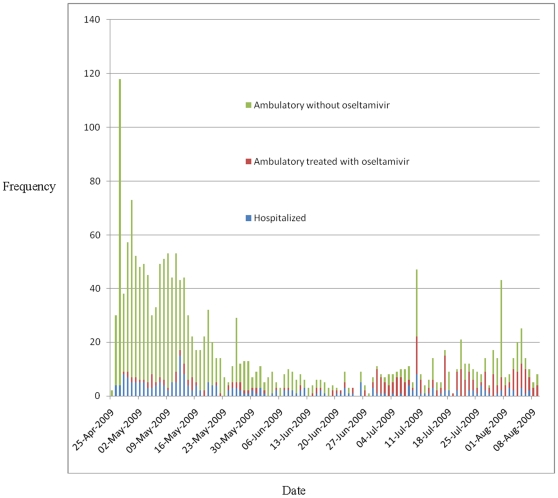
Histogram of patients seeking care for acute respiratory infections at Hospital Civil de Guadalajara during the (H1N1) pandemic 2009—Mexico.

**Figure 3:**
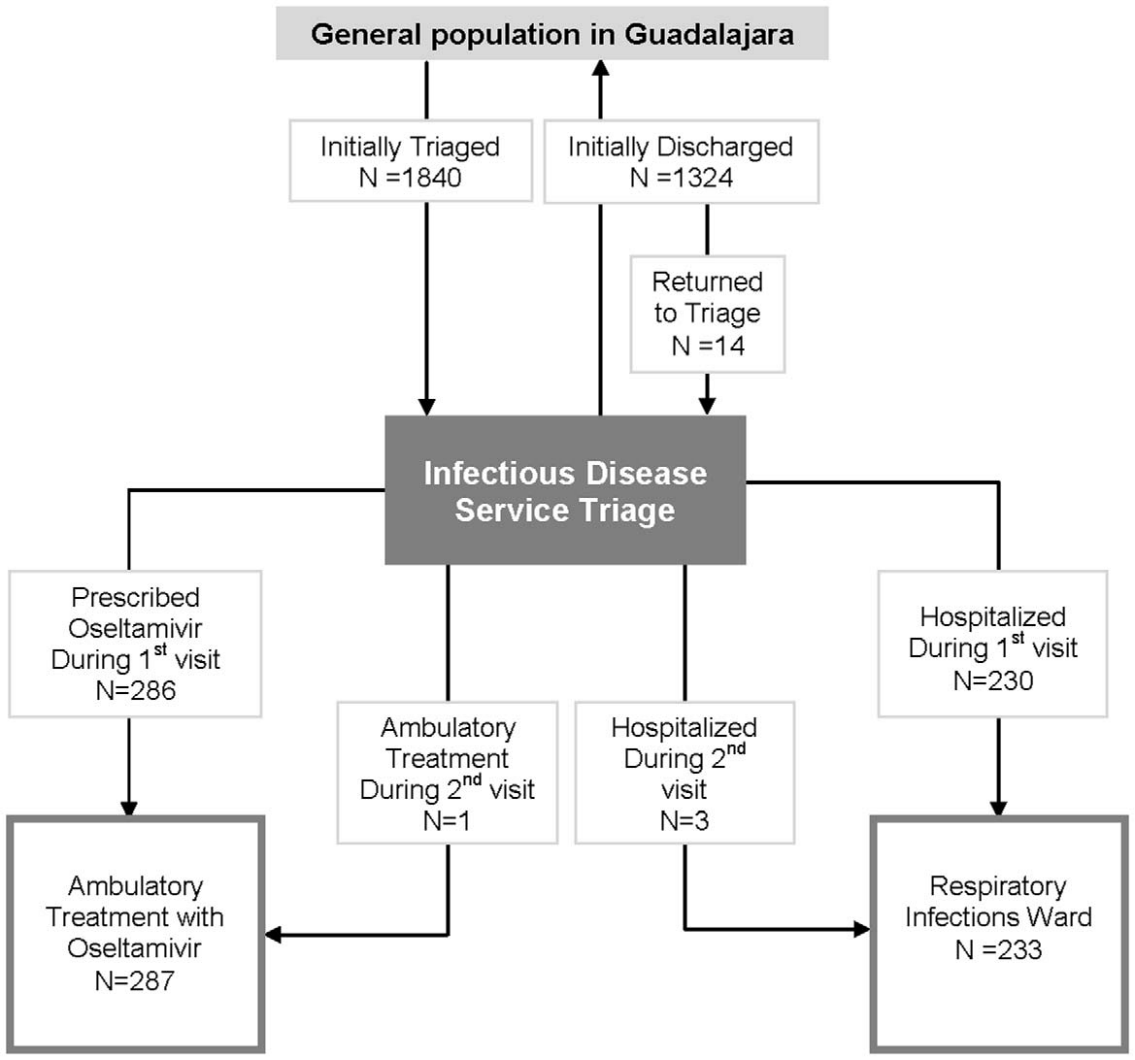
Patients seeking care with acute respiratory infections at the Hospital Civil de Guadalajara during the (H1N1) pandemic 2009—Mexico.

During April 25–August 9, hospital staff triaged 1840 persons with acute respiratory infections (Figure 2). Patients' median age was 29 years [IQR 22–41 years] and 55% were female. Of the 1840 ER patients, 167 (9.1%) were classified at high risk (mean ILI-score = 19), 725 (39.4%) at intermediate risk (median ILI-score = 10), and 945 (51.4%) at low risk (median ILI-score = 3) of developing complications of presumptive 2009 pandemic influenza A (H1N1) disease (Table 1). Two-hundred and thirty (12.5%) were admitted to hospital (median ILI-score = 15 [IQR = 11–19]) (Figure 3). Of 286 ambulatory patients who were prescribed oseltamivir (median ILI-score = 11, IQR = 7–15), none required subsequent medical evaluation. Of 1324 ambulatory patients who were not treated with oseltamivir (median ILI-score = 5, IQR = 1–8), 14 (0.8%) returned a median of 8 days after their initial visit. Three (21%) of the 14 returning patients (i.e. one pregnant and two with a history of tobacco abuse), were hospitalized and treated with oseltamivir (with a median ILI-score  = 19). Two of these 3 returning patients who were subsequently hospitalized tested positive for 2009 pandemic influenza (H1N1). One (7%) of the 14 returning patients was prescribed oseltamivir and discharged from the ED, and 10 (71%) were discharged home without oseltamivir. One patient visited triage three times, but was not treated with oseltamivir. Three deaths occurred in hospitalized patients (aged 18, 37, and 54 years). Decedents presented to the ER a mean of 4 days after symptom onset with a mean ILI score of 16. One decedent was confirmed with pandemic H1N1, one had seasonal influenza A, and one was not tested. All other hospitalized patients improved and were discharged home.

**Table 1:** Demographic Characteristics of Patients Seeking Care for acute respiratory infection at the Hospital Civil de Guadalajara during the (H1N1) pandemic 2009—Mexico.

Demographics N (%)	All initially triaged patients (N = 1840)	All hospitalized patients treated with oseltamivir (N = 233)‡	All ambulatory patients treated with oseltamivir as outpatients (N = 286) ‡	Patients discharged from triage without oseltamivir (N = 1324)	All patients treated with oseltamivir with seasonal influenza A cases (N = 42)∞	All patients treated with oseltamivir with pandemic (H1N1) 2009 cases (N = 104) ∞
Median age	29	28	29	29	31	23*
Females	1017 (55%)	134 (58%)	154 (54%)	741 (55%)	20 (48%)	45 (43%)
**Most Frequently Reported Occupations**						
Home makers	376 (20%)	62 (27%)	32 (11%)	287 (22%)	8 (19%)	12 (12%)
Students	288 (16%)	40 (17%)	51 (18%)	198 (15%)	3 (7%)	28 (28%)
Health care workers	230 (13%)	17 (7%)	88 (31%)	126 (10%)	6 (14%)	13 (12%)
Retail workers	163 (9%)	18 (8%)	14 (5%)	132 (10%)	4 (10%)	3 (3%)
Construction workers	121 (7%)	8 (3%)	4 (1%)	111 (8%)	2 (5%)	5 (4%)
Unemployed	74 (4%)	11 (5%)	4 (1%)	60 (5%)	3 (7%)	1 (1%)
Assessment of risk						
High risk	167 (9%)	114 (49%)	52 (18%)	4 (0.3%)	14 (33%)	38 (37%)
Intermediate risk	725 (39%)	104 (45%)	173 (60%)	451 (34%)	18 (43%)	49 (47%)
Low risk	945 (51%)	15 (6%)	59 (21%)	880 (66%)	10 (24%)	14 (16%)¥
Median ILI-score	6	15	11	5	14	13

*Difference between seasonal influenza and pandemic (H1N1), 2009, p = 0.0007.

¥2% of pandemic (H1N1) 2009 missing risk assessment information.

‡Includes all hospitalized cases regardless of influenza RT-PCR test results.

∞Includes all hospitalized cases and ambulatory patients treated with oseltamivir who tested positive for influenza A.

### Characteristics of hospitalized patients

Hospitalized patients presented within a median of 2 days after symptom onset with dyspnea and abnormal findings on chest imaging. Sixty-seven (30%) of the 230 hospitalized patients smoked tobacco (for a mean duration of 8 years), 45 (20%) had a history of alcohol abuse (i.e. using CAGE questionnaire), and 22 (10%) had a history of other drug use (Table 2). Ninety-one percent of hospitalized patients reported fatigue, 90% headache, 88% myalgias, 86% fever, 82% chills, and 63% dry cough (Table 3). During triage, fever (i.e. measured temperature ≥38°C) was documented in 184 (79%) of the 233 hospitalized patients (Table 4). Sixteen (33%) of the 49 hospitalized patients who were afebrile at triage reported using paracetamol, non-steroidal anti-inflammatory medications or oral corticosteroids prior to their ER visit. Nine (4%) of the 233 hospitalized patients had hypoxia (i.e. PO_2_ <70), 4 had hypotension (blood pressure <90/60), and 3 required invasive mechanical ventilation. One-hundred and fifty-six (69%) of 233 hospitalized patients had lymphopenia compared to 117 (41%) of 286 ambulatory patients treated with oseltamivir (p<0.0001). Similarly, 35 (15%) of 233 hospitalized patients had thrombocytopenia compared to 19 (7%) of 286 ambulatory patients treated with oseltamivir (p<0.001). Out of the 181 hospitalized patients tested, 36 (20%) were positive for 2009 pandemic influenza A (H1N1) and 24 (13%) were positive for seasonal influenza A. Similarly, out of the 187 hospitalized patients tested, 68 (36%) were positive for 2009 pandemic influenza A (H1N1) and 18 (10%) were positive for seasonal influenza A.

**Table 2:** Symptoms of Patients Seeking Care for Acute Respiratory Infections at the Hospital Civil de Guadalajara during the (H1N1) pandemic 2009—Mexico_◊_.

All initially triaged patients (N = 1239)	All hospitalized patients treated with oseltamivir (N = 233)‡	All ambulatory patients treated with oseltamivir as outpatients (N = 286) ‡	All patients treated with oseltamivir with seasonal influenza A cases (N = 42) ∞	All patients treated with oseltamivir with pandemic (H1N1) 2009 cases (N = 104) ∞
**Past medical history** N (%)				
Smoking	67 (30%)	4 (1%)	8 (19%)	13 (12%)
Alcoholism	45 (20%)	6 (2%)	6 (14%)	10 (10%)
Drug abuse	22 (10%)	0 (0%)	4 (10%)	3 (3%)
Hypertension	20 (9%)	5 (2%)	3 (7%)	4 (4%)
Diabetes	13 (6%)	7 (2%)	4 (10%)	2 (2%)
Tuberculosis	11 (5%)	1 (0.3%)	2 (5%)	1 (1%)
Asthma	9 (4%)	5 (2%)	1 (2%)	1 (1%)
Other lung disease	9 (4%)	3 (1%)	2 (5%)	1 (1%)
Other immune suppression	8 (4%)	5 (2%)	1 (2%)	2 (2%)
Neurological disease	5 (2%)	1 (0.3%)	1 (2%)	1 (1%)
Chronic renal problems	5 (2%)	2 (1%)	2 (5%)	2 (2%)
HIV	4 (2%)	1 (0.3%)	0 (0%)	0 (0%)
Pregnancy	3 (2%)	1 (0.3%)	0 (0%)	0 (0%)
Obesity	3 (1%)	0 (0%)	0 (0%)	1 (1%)
Malnutrition	2 (1%)	0 (0%)	0 (0%)	1 (1%)
Transplant	2 (1%)	1 (0.3%)	0 (0%)	1 (1%)
Influenza vaccine	31 (13%)	52 (18%)	8 (19%)	13 (12%)

‡Includes all hospitalized cases regardless of influenza RT-PCR test results.

∞Includes all hospitalized cases and ambulatory patients treated with oseltamivir who tested positive for influenza A.

◊Insufficient data available from patients discharged from triage without oseltamivir to include in table.

**Table 3:** Presenting Symptoms of Patients Seeking Care for Acute Respiratory Infections at the Hospital Civil de Guadalajara during the (H1N1) pandemic 2009—Mexico.

	All initially triaged patients (N = 1840)	All hospitalized patients treated with oseltamivir (N = 233)‡	All ambulatory patients treated with oseltamivir as outpatients (N = 286) ‡	Patients discharged from triage without oseltamivir (N = 1324)	All patients treated with oseltamivir with seasonal influenza A cases (N = 42) ∞	All patients treated with oseltamivir with pandemic (H1N1) 2009 cases (N = 104) ∞
**Symptoms** N (%)						
Median symptom onset before presentation	2d	2d	2d	2d	2d	2d
Headache	1460 (79%)	210 (90%)	249 (87%)	10111 (76%)	32 (76%)	93 (88%)*
Myalgia	1336 (73%)	204 (88%)	224 (78%)	919 (69%)	31 (74%)	85 (81%)
Fatigue	1254 (68%)	212 (91%)	228 (79%)	829 (62%)	33 (79%)	88 (83%)
Sore throat	1251 (68%)	163 (70%)	192 (67%)	906 (68%)	28 (67%)	75 (70%)
Chills	1087 (59%)	190 (82%)	203 (71%)	704 (53%)	32 (76%)	76 (74%)
Dry cough	951 (52%)	147 (63%)	172 (60%)	637 (48%)	23 (55%)	69 (64%)
Subjective Fever	888 (48%)	201 (86%)	203 (71%)	492 (37%)	33 (79%)	90 (85%)
Conjunctivitis	791 (43%)	127 (55%)	115 (40%)	556 (42%)	22 (52%)	48 (46%)
Rhinorrhea	637 (35%)	100 (43%)	153 (53%)	387 (29%)	17 (40%)	53 (51%)
Thoracic pain	561 (30%)	130 (56%)	109 (38%)	329 (24%)	24 (57%)	49 (45%)
Productive cough	492 (27%)	47 (20%)	66 (23%)	381 (29%)	8 (19%)	32 (31%)
Dyspnea	438 (24%)	120 (52%)	90 (31%)	230 (17%)	13 (31%)	42 (40%)
Diarrhea	244 (13%)	56 (24%)	57 (20%)	132 (10%)	7 (17%)	21 (20%)
Abdominal pain	240 (13%)	52 (23%)	56 (20%)	132 (10%)	7 (17%)	21 (20%)
Rales	37 (2%)	33 (14%)	4 (1%)	0 (0%)	4 (10%)	3 (3%)
Wheezing	14 (1%)	13 (6%)	1 (0.3%)	0 (0%)	0 (0%)	1 (1%)

*p = 0.04 when comparing pandemic (H1N1) 2009 test positives to seasonal influenza A test positives.

‡Includes all hospitalized cases regardless of influenza RT-PCR test results.

∞Includes all hospitalized cases and ambulatory patients treated with oseltamivir who tested positive for influenza A.

**Table 4:** Findings of patients seeking care for acute respiratory infections at the Hospital Civil de Guadalajara during the (H1N1) pandemic 2009—Mexico _◊_.

	All hospitalized patients treated with oseltamivir (N = 233)‡	All ambulatory patients treated with oseltamivir as outpatients (N = 286) ‡	All patients treated with oseltamivir with seasonal influenza A cases (N = 42) ∞	All patients treated with oseltamivir with pandemic (H1N1) 2009 cases (N = 104) ∞
**Findings**				
Median temperature (°C)	38.5	37.7	38.5	38
Hypoxia N (%)	9 (5%)	0 (0%)	1 (2%)	1 (1%)
Lymphopenia	156 (69%)	117 (41%)	27 (64%)	66 (63%)
Thrombocytopenia	35 (15%)	19 (7%)	2 (5%)	9 (9%)
**Radiology** N (%)	Of 205 hospitalized patients who had chest X-ray [of which 83 had chest CT]	Of 258 ambulatory patients who had chest X-ray [of which 35 had chest CT]	Of 36 patients who tested positive for seasonal influenza A and who had chest X-ray s[of which 16 had chest CT]	Of 95 patients who tested positive for pandemic (H1N1) and who had chest X-rays [of which 30 had chest CT]
Abnormal chest X-ray	79 (39%)	112 (43%)	14 (39%)	59 (62%)¥
Abnormal lung CT	91 (97%)	30 (86%)	16 (100%)	30 (100%)
Bilateral infiltrates	49 (53%)	23 (64%)	12 (75%)	28 (93%)*
Tree-in bud appearance	69 (73%)	26 (72%)	15 (94%)	28 (93%)*
Involvement of basal zone	62 (66%)	24 (67%)	16 (100%)	30 (100%)*
Air trapping	52 (55%)	23 (64%)	15 (94%)	27 (90%)*
Centrilobular nodules	49 (52%)	21 (58%)	13 (81%)	24 (80%)*
Thickened interlobar septa	48 (51%)	21 (58%)	12 (75%)	28 (93%)*
Multifocal distribution	38 (40%)	13 (36%)	11 (69%)	19 (63%)*
Involvement of middle zone	27 (29%)	9 (25%)	6 (37%)	18 (60%)*
Segmental consolidation	15 (16%)	1 (3%)	1 (6%)	5 (17%)
Segmental distribution	14 (15%)	0 (0%)	1 (6%)	3 (10%)
Involvement of apical zone	10 (11%)	4 (11%)	1 (6%)	3 (10%)
Peribronchial ground glass	7 (7%)	4 (11%)	1 (6%)	7 (23%)*

¥p = 0.01 when comparing patients who tested positive for seasonal influenza A with those who tested positive for pandemic (H1N1) 2009.

*p≤0.009 when comparing patients who tested positive for pandemic (H1N1) 2009 to those who tested negative.

‡Includes all hospitalized cases regardless of influenza RT-PCR test results.

∞Includes all hospitalized cases and ambulatory patients treated with oseltamivir who tested positive for influenza A.

◊Insufficient data available from patients discharged from triage without oseltamivir to include in table.

### Clinical presentation of patients who tested positive for 2009 pandemic influenza A (H1N1) virus

**Figure 4:**
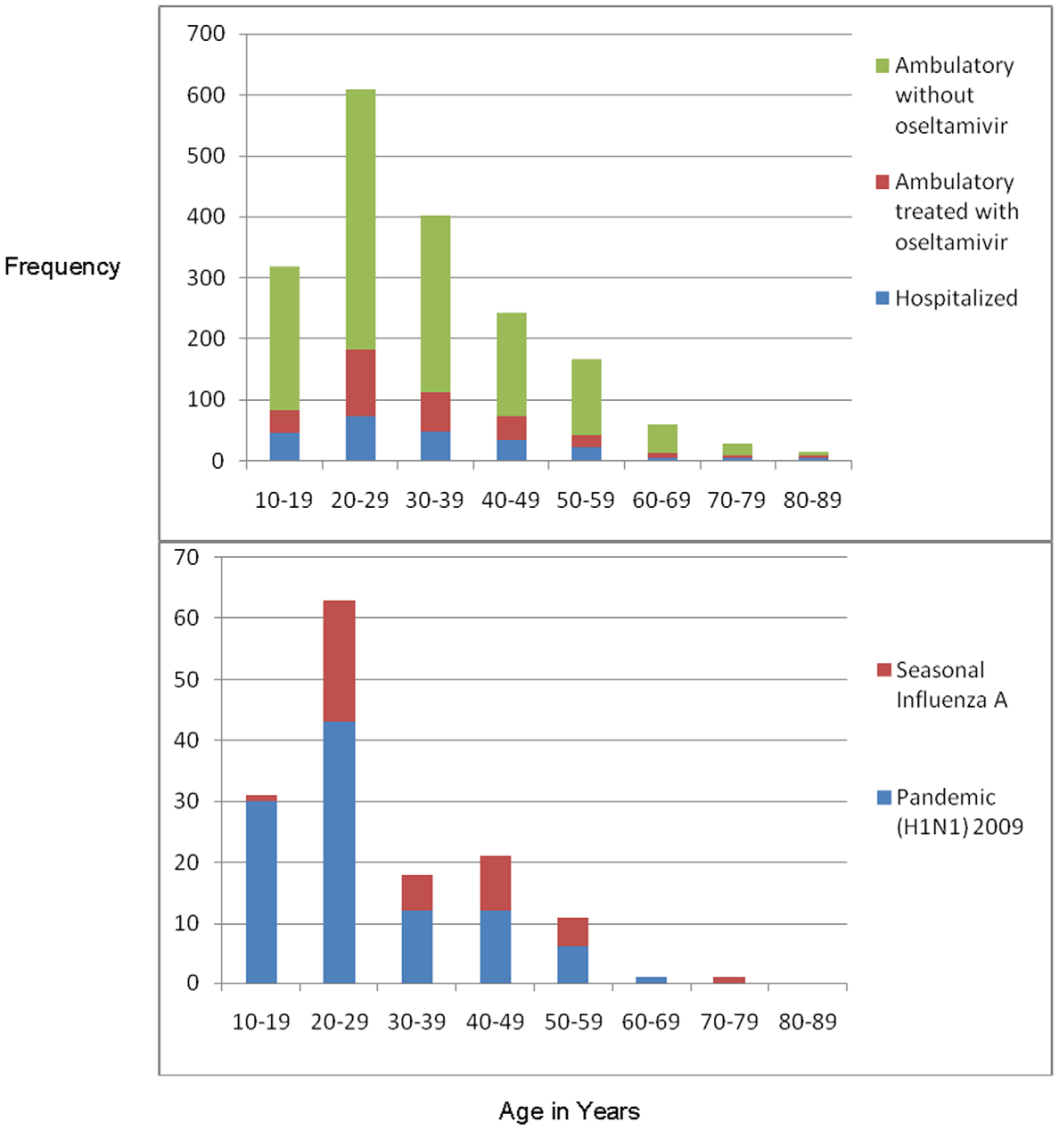
Age distribution of patients triaged for acute respiratory infections at the Hospital Civil de Guadalajara during the (H1N1) pandemic 2009—Mexico.

Of the 1840 persons triaged, 379 (21%) were tested for influenza (i.e. 371 (20%) by rRT-PCR, 112 (6%) by rapid diagnostic test, and 89 (5%) by immunofluorescence). Of the 371 patients tested by rRT-PCR, 104 (28%) had pandemic (H1N1) and 42 (11%) had seasonal influenza A detected. There was a 0.51 correlation between rRT-PCR and rapid diagnostic test results among the 85 patients who were tested by both methods (p<0.001). In contrast, there was a 0.15 correlation between rRT-PCR and immunofluorescence results among the 57 who were tested by both methods. In comparison to patients with seasonal influenza, patients whose rRT-PCR tested positive for 2009 pandemic influenza A (H1N1) were younger (Figure 4). The median age of patients who tested positive for 2009 pandemic influenza A (H1N1) was 23 years versus 31 years for patients who tested positive for seasonal influenza A (p = 0.0007)(Table 1). Patients whose rRT-PCR tested positive for 2009 pandemic influenza A (H1N1) were not more likely to be pregnant, report substance abuse, have other medical conditions (e.g. obesity), or require hospitalization within 2 days of developing symptoms than other patients (Table 2). At ER presentation, 69 (66%) of the 104 patients whose rRT-PCR tested positive for 2009 pandemic influenza A (H1N1) reported a dry cough (mean duration  = 3 days) versus 145 (55%) of 264 test negative patients (p = 0.03). Thirty-two (31%) the 104 patients whose rRT-PCR tested positive for 2009 pandemic influenza A (H1N1) had a productive cough compared to 53 (20%) of 262 test negative patients (p = 0.03). Patients whose rRT-PCR tested positive for 2009 pandemic influenza A (H1N1) presented with a median temperature of 38.5°C which, on average, started 2 days before admission [IQR1–3]. There were no differences in WBC at ER presentation between patients whose rRT-PCR tested positive for 2009 pandemic influenza A (H1N1) and patients who tested negative for 2009 pandemic influenza A (H1N1).

### Radiological findings of hospitalized patients

**Figure 5:**
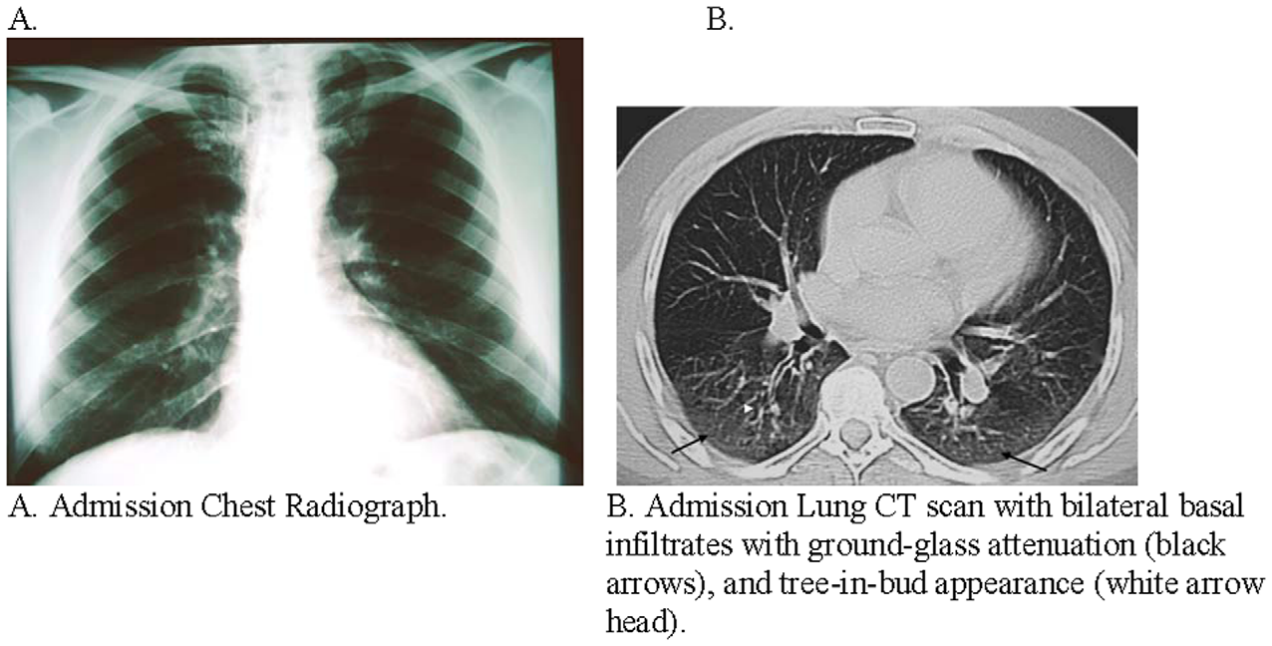
Typical radiological findings of Pandemic (H1N1) 2009 patient at the Hospital Civil de Guadalajara—Mexico.

Eighteen (60%) of 30 hospitalized patients infected with 2009 pandemic influenza A (H1N1) with chest X-rays had abnormal findings while all 22 with chest CT scans had abnormal findings (Table 4). Similarly, 5 (25%) of 20 hospitalized patients infected with seasonal influenza A who had chest X-rays had abnormal findings while all 10 who had chest CT had abnormal findings. Hospitalized patients infected with 2009 pandemic influenza A (H1N1) were more likely to have abnormal chest X-rays than patients infected with seasonal influenza A (p = 0.02) (Table 4). Twenty (91%) of 22 imaged hospitalized patients infected with 2009 pandemic influenza A (H1N1) had bilateral infiltrates on chest X-ray or CT compared to 23 (38%) of 61 imaged patients who tested negative for 2009 pandemic influenza A (H1N1)(p<0.001). Similarly, more patients infected with 2009 pandemic influenza A (H1N1) had chest X-rays and CT scans with thickened interlobar septa (p<0.001), involvement of the middle zone (p<0.001), compared to imaged patients who tested negative for 2009 pandemic influenza A (H1N1) (Table 4) (Figure 5).

### Risk factors for increased length of hospitalization among 2009 pandemic influenza A (H1N1) cases

Testing positive for 2009 pandemic influenza A (H1N1) was not associated with prolonged stay. On average, 2009 pandemic influenza A (H1N1) patients were hospitalized for a median of 2 days [IQR 1–3days]. 2009 Pandemic influenza A (H1N1) infected patients with dyspnea on admission had a mean hospital stay of 2.1 days while those without dyspnea had a mean hospital stay of 1.3 days. The one decedent infected with pandemic influenza A (H1N1) presented 6 days after symptom onset with dyspnea and a 10 year history smoking history. There were no reported adverse events among patients associated with the use of oseltamivir.

## Discussion

During 6 weeks when there was co-circulation of pandemic and seasonal influenza A viruses in the community, hospital staff triaged more than eighteen-hundred patients with respiratory complaints and identified 12% for inpatient care. The triage system was based on assumptions about who is at risk for developing complications from seasonal influenza (e.g. patients aged over 65 years). Our analyses, however, suggested that patients infected with 2009 pandemic influenza A (H1N1) tended to be younger than seasonal influenza A patients. Nevertheless, our data suggest that clinicians used the ILI-score to help them determine, with minimal misclassification, which patients needed hospitalization versus who could be managed as outpatients [18]. The ILI-score helped guide clinicians to decide who needed hospital care and antiviral treatment when timely laboratory confirmation of influenza was not available. Only 1% of patients triaged needed re-evaluation. Such a system could be readily used to efficiently triage patients during outbreaks and epidemics by adapting the system's scores to match the anticipated characteristics of patients who are at highest risk of developing complications.

While the triaging system led clinicians to hospitalize traditional groups at risk for complications from seasonal influenza (i.e. those with chronic medical illnesses), patients infected with 2009 pandemic influenza A (H1N1) were often young and had few pre-existing conditions [20]. These data are comparable with Mexican Directorate General of Epidemiology data that suggest 56% of pandemic (H1N1) confirmed deaths occurred among those aged 30–59 years, many of whom were previously healthy [21]. The age shift in 2009 pandemic influenza A (H1N1) cases may be caused by cross-reactive immunity from prior influenza infections in 33% of those aged more than 60 years [22], [23]. Health officials should adjust pandemic triaging tools to account for the younger age distribution of cases [24]. Pregnancy should also be included as a risk factor in triaging tools. Although there were too few pregnant women in our case series for subgroup analyses, other data suggest pregnant women are at high risk of developing severe complications from 2009 pandemic influenza A (H1N1) [25].

In this case series, hospitalized patients who tested positive for 2009 pandemic influenza A (H1N1) received oseltamivir within 2 days of symptom onset and appeared to recover quickly with a median hospital stay of two days. Similarly, no ambulatory patients treated with oseltamivir required further medical care. In contrast, 3 patients initially discharged from the ED without oseltamivir returned to triage and required hospital admission. Two of these 3 later tested positive for 2009 pandemic influenza A (H1N1). One additional patient who required mechanical ventilation and subsequently died had presented 6 days after symptom onset. Another 5 hospital decedent whose care was transferred to the infectious disease service and therefore not part of our triaged case-series presented a median of 15 days after symptom onset. These cases suggest the importance of early oseltamivir treatment.

Only one (1%) of 104 patients who tested positive for 2009 pandemic influenza A (H1N1) case-patients died. These findings contrast those of the National Institute of Respiratory Diseases in Mexico City where 12 (67%) of 18 patients required mechanical ventilation and 7 (39%) patients died [24], [26]. The discrepancy between these two case-series may be explained by when the populations served by these hospitals were affected by the pandemic. National Institute of Respiratory Diseases data were collected during March 24–April 24, 2009, when it was still unclear that a proportion of cases with severe acute respiratory infections had 2009 pandemic influenza A (H1N1). In Guadalajara, the outbreak started later. Hospitalized patients we described received earlier oseltamivir. Our patients were hospitalized during April 25–August 9. Seventy-five percent of our case-patients received oseltamivir within 72 hours of symptom onset. Patients in the Mexico City case-series presented with severe disease an average of 8 days after illness onset and received late oseltamivir.

Our findings have important limitations. A minority of all patients had respiratory specimens tested by RT-PCR, a large number of patients who were triaged were not confirmed with seasonal influenza or 2009 pandemic influenza A (H1N1) virus infection. No testing for other etiologies of acute respiratory illness was performed. Oseltamivir treatment among hospitalized patients was not randomized among cases and control. No comparison group was available to assess oseltamivir effectiveness for the treatment of 2009 pandemic influenza A (H1N1).

The triaging system with its ILI-score needs further validation. Nevertheless, such a triaging system can help guide the clinical management of patients presenting to the ED with acute respiratory illness in settings that lack timely diagnostic testing and have limited antivirals supplies. With some adaptation, the system may be especially useful in resource-poor countries, during the peak of pandemic influenza, or during other respiratory virus activity. Although no scoring system will replace clinical judgment, our experience suggests that the triaging system may have helped clinicians effectively triage patients and determine who needed hospital care and who could be managed as outpatients. The triaging system and the ILI-score should be modified to the local 2009 pandemic influenza A (H1N1) situation based upon hospital surge capacity, antiviral susceptibilities and supply, and the evolving epidemiology of this virus.
